# Evaluation of changes in mean choroidal thickness before and after treatment with steroids and laser photocoagulation in patients with Eales’ disease

**DOI:** 10.1186/s40942-025-00731-z

**Published:** 2025-10-27

**Authors:** Meenakshi Thakar, Obinam Muang, Paromita Dutta, Bhumika Sharma

**Affiliations:** Guru Nanak Eye Centre, Maulana Azad Medical College, New Delhi, India

**Keywords:** Eales’ disease, Mean choroidal thickness, Spectral domain optical coherence tomography, Enhanced depth imaging optical coherence tomography, Laser photocoagulation, Periphlebitis

## Abstract

**Background:**

Eales’ disease is an idiopathic peripheral retinal phlebitis, which is conventionally believed to be limited to the retinal layers. Recent research has shown additional involvement of the choroid, too. Spectral domain-optical coherence tomography (SD-OCT) with enhanced depth imaging (EDI-OCT) allows detailed visualization of the choroid. This study was done to evaluate the change in mean choroidal thickness (MCT) pre- and post-treatment with laser photocoagulation, in patients of Eales’ disease using SD-OCT with EDI.

**Methods:**

Twenty eyes of 20 male patients with Eales’ disease (cases) and twenty eyes of 20 age and sex-matched controls were included. EDI-OCT was used to measure choroidal thickness (from the outer border of retinal pigment epithelium to choroid-sclera junction at fovea, and at 500 μm intervals up to 1500 μm temporal and nasal to fovea at 7 locations), and central macular thickness (CMT), in both groups. MCT was calculated as the mean of the seven values. Cases were treated with oral steroids followed by frequency-doubled Nd-Yag multispot retinal laser photocoagulation. MCT was compared at baseline, 6 weeks, and 3 months after laser.

**Results:**

MCT of Eales’ patients were greater and significantly different as compared to the control group at baseline (338.80 ± 46.74 μm vs. 299.80 ± 20.69 μm, *p* < 0.01), 6 weeks (343.25 ± 44.79 μm vs. 299.60 ± 20.05 μm, *p* < 0.01), and 3 months after laser (353.25 ± 29.25 μm vs. 297.20 ± 24.33 μm, respectively, *p* < 0.01). The increase in MCT after treatment was not statistically significant. Best corrected visual acuity (BCVA) did not change significantly from baseline after treatment. CMT of the two groups was comparable at baseline (240.5 ± 30.90 μm vs. 253.40 ± 23.52 μm, *p* = 0.10), 6th week (258.20 ± 36.44 μm vs. 247.25 ± 22.79 μm, *p* = 0.43), and 3rd month (247.40 ± 32.25 μm vs. 249.65 ± 24.01 μm, *p* = 0.60).

**Conclusion:**

The MCT in Eales’ patients is significantly higher than that of normal healthy controls at presentation. Laser photocoagulation does not decrease the MCT in the short term, up to 3 months. The increased MCT does not affect the BCVA in the short term.

## Background

Eales’ disease is an idiopathic inflammatory venous occlusive disease that primarily affects the peripheral retina of young adult males. The condition is characterized by three stages: vascular inflammations, mainly periphlebitis, vascular occlusion, and retinal neovascularization, along with sequelae of complications. While the inflammatory phase is managed with steroids, photocoagulation of the ischemic retina is essential to curtail the stimulus for neovascularization [1]. The disease is also characterized by recurrent vitreous hemorrhages [1]. Contrary to the common perception that the pathology is confined to the retinal layers, lymphocytic infiltration in the iris, ciliary body, and vitreous has been demonstrated in enucleated globes with Eales’ disease [2, 3]. In vivo imaging of the choroid in patients with active disease, using spectral domain optical coherence tomography (SD-OCT) with enhanced depth imaging (EDI), revealed increased sub-foveal choroidal thickness (CT) [4]. Since one of the purported mechanisms of Eales’ disease is an immunological response to an exogenous stimulus, the involvement of the highly vascular choroid is explainable, whether as a part of the inflammation or in response to the elevated levels of vascular endothelial growth factors. CT has been extensively studied in other posterior uveitic/ panuveitic conditions of Vogt-Koyanagi-Harada syndrome, Behçet’s disease, and birdshot retinochoroiditis, where disease activity and duration correlated with CT. CT has been observed to decrease with systemic steroids and activity quiescence [5]. CT changes have also been documented in conditions with systemic vasculitis and may be considered as prognostic biomarkers [6]. CT in Eales’ disease has not been studied in detail, possibly due to its prevalence in certain areas only. This study was conducted to evaluate and compare the changes in mean choroidal thickness (MCT) and central macular thickness (CMT) in eyes with Eales’ disease before and after treatment with laser photocoagulation and oral steroids.

## Methods

This was a prospective, interventional study conducted over a period of one year at a tertiary care centre in northern India. The study protocol received approval from the institutional ethics committee (F.1/IEC/MAMC/MD/MS (96/02/2023/No.227)), and all procedures were performed in adherence to the ethical principles outlined in the Declaration of Helsinki. Written informed consent was obtained from all participants before enrolment in the study, ensuring their voluntary participation and understanding of the study’s objectives and procedures.

Patients with peripheral vasculitis were screened. Diagnosis of Eales’ disease was based on fundus findings of peripheral occlusive vasculitis of idiopathic origin, with or without spontaneous, recurrent vitreous hemorrhage in healthy young patients. All patients underwent a detailed vasculitis work-up to exclude systemic associations and ocular conditions causing similar presentation, such as diabetic retinopathy, venous occlusion, raised serum homocysteine levels, sarcoidosis, syphilis, blood dyscrasias, Coats disease, familial exudative vitreoretinopathy, acquired immunodeficiency, Behcet’s disease, and sickle-cell disease. Blood investigations included erythrocyte sedimentation rate, complete blood count, peripheral smear, glycosylated hemoglobin, serology for human immunodeficiency virus type 1 and 2, Hepatitis C Virus, Hepatitis B surface antigen, venereal disease research laboratory test, serum angiotensin converting enzyme levels, and antinuclear antibody. Chest X-ray and Mantoux test were performed to rule out tuberculosis. High-resolution computerized tomography of the chest was advised in patients with clinical and radiological suspicion of tuberculosis. The pulmonologist made the final decision regarding the need for initiating anti-tubercular therapy (ATT). Ophthalmic examination included best corrected visual acuity (BCVA), slit lamp biomicroscopy, and fundus examination. Patients with vascular sheathing, perivenous exudates, and superficial retinal hemorrhages were classified as active vasculitis, while those with sclerosed vessels were diagnosed as healed vasculitis. Fundus fluorescein angiography (FFA, Visucam Carl Zeiss Meditec, Germany) was performed in all study patients to assess the severity of retinal capillary non-perfusion, the presence of neovascularization, and as a guide for laser photocoagulation [7]. The stage of Eales’ disease was classified according to the classification by Saxena et al. [8].

Inclusion criteria for the study were treatment-naive patients with Eales’ disease, between 16 and 45 years of age, grade IIA–IIIa retinopathy, with adequate media clarity for good quality FFA and spectral domain optical coherence tomography (SD-OCT) with enhanced depth imaging (EDI). The exclusion criteria were any other retinal diseases, glaucoma, prior retinal laser, intraocular surgery, axial length > 26 mm and < 22 mm, history of immunosuppressive therapy, and smoking.

All eyes underwent OCT with EDI to assess mean choroidal thickness (MCT) and central macular thickness (CMT), using SD-OCT (Carl Zeiss Meditec, Inc., Dublin, CA, USA). The fovea was scanned with a 1-line raster, a 6-mm line consisting of 4096 A-scans. Choroidal thickness was measured by using the calliper function from the outer border of the retinal pigment epithelium (RPE) to the choroid-sclera junction at the fovea and at 500 μm intervals up to 1500 μm temporal as well as nasal to the fovea at 7 locations. The MCT (mean choroidal thickness) was calculated as the arithmetic mean of the 7 measurements [9]. All measurements were performed by two masked observers who were not aware of the retinal status of the subjects, and the average of the two MCT readings was taken. All readings were obtained between 09:00 am and 11:00 am to avoid diurnal variation in MCT.

### Treatment protocol

All study patients received treatment according to the standard of care protocol [1]. All patients received prednisolone (dose of 1 mg/kg body weight) for active vasculitis, which was tapered by 5 to 10 mg per week over 4–6 weeks (mean 4.3+/-0.23 weeks). Sectoral or pan retinal photocoagulation (PRP) with frequency-doubled Nd-Yag multispot retinal laser was then performed, depending on capillary non-perfusion (CNP) area and presence of neovascularization elsewhere (NVE).

MCT was evaluated at baseline, 6 weeks, and 3 months after initiating treatment.

### Controls

Age and sex-matched healthy controls were selected, using similar exclusion criteria as for cases. All controls underwent a detailed ophthalmic work-up. The timeframe for MCT assessment was the same as that for cases.

### Statistical analysis

The collected data was entered in Microsoft Excel and statistically analysed using SPSS-PC-25 version. Quantitative data was expressed as mean with standard deviation or median with interquartile range, depending on the distribution. Normality of variables was assessed by using the Kolmogorov-Smirnov test. The MCT and CMT were normally distributed. An unpaired t-test was used for intergroup comparison, while Repeated Measures ANOVA was used for intragroup comparison at different time intervals, followed by a post hoc test. The difference between two means for data that was not normally distributed (age, BCVA) was tested by the Mann-Whitney U test, while for before and after treatment comparison, the Wilcoxon signed-rank test was used. Qualitative data were expressed in percentages, and the difference between the proportions was tested by the chi-square test or Fisher’s exact test. A p-value less than 0.05 was considered statistically significant.

## Results

The study included 20 eyes of patients with Eales’ disease (Case group) and 20 eyes of healthy individuals as the control group. The baseline characteristics of cases and controls are summarized in Table 1. The mean age of patients in both groups was comparable. A subgroup analysis of age distribution showed that even though 8 cases were in the 31–40 years group, there were no controls in the same age group. A relook at the age data showed that the oldest amongst these patients (Cases) was 31.5 years old. All the patients in both groups were males.

**Table 1:** Baseline characteristics of cases and controls

	Cases (*n* = 20)	Controls (*n* = 20)	*p*-value
Mean age in years	28.80 ± 6.28	26.50 ± 1.60	0.12^#^
18–30 years	12 (60%)	20 (100%)	
31–40 years	8 (40%)	0	
Mean axial length	22.92 + 0.13	23.21 + 0.42	0.493
Mean spherical equivalent refractive error (in diopters)	-0.966 *±* 1.073	-0.981 *±* 1.126	0.931^#^
Mean visual acuity (logMar)	0.08 ± 0.11	0.04 ± 0.08	0.23^#^

^#^ Mann-Whitney U test

Only one patient had bilateral involvement at presentation. However, since one eye had a vitreous hemorrhage, only the other eye was included in the study. No study patient was found to have evidence of active tuberculosis. The average duration of oral prednisolone and time for clinical resolution of the inflammation was 4.3 *±* 0.8 weeks. None of the patients had active tuberculosis and hence did not receive anti-tubercular therapy.

The MCT values showed an intraclass correlation coefficient (ICC of 0.973) between the two observers and an intra-observer agreement ICC of 0.965, both indicating consistency of measurement.

**Fig. 1:**
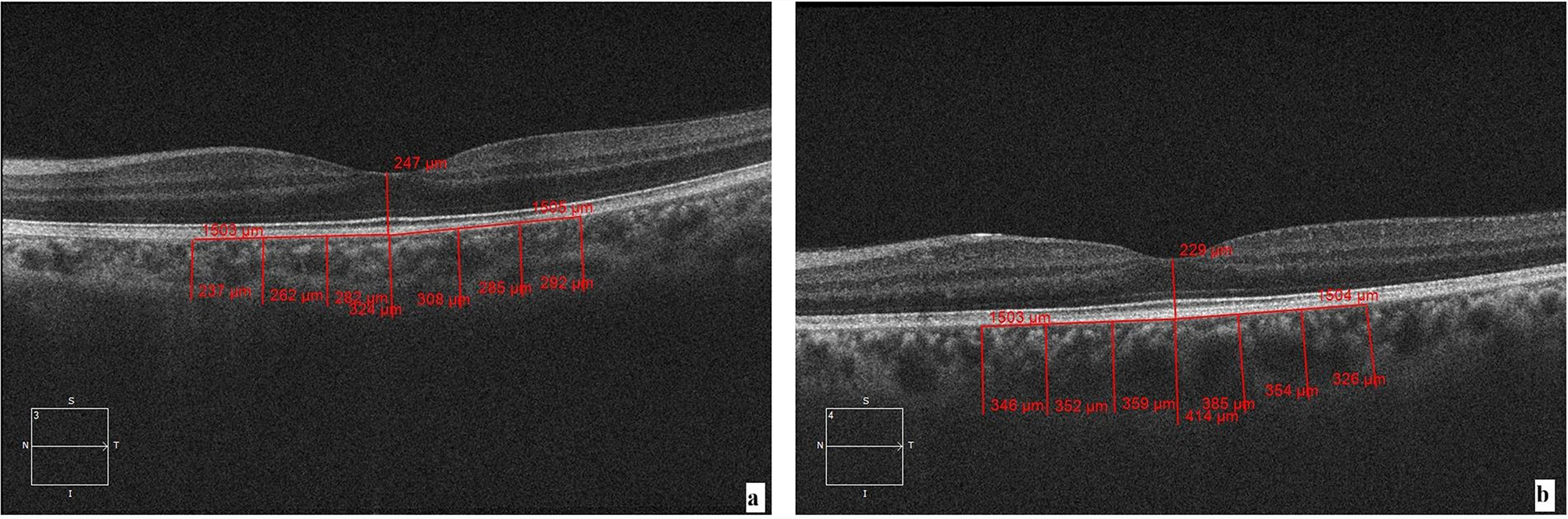
Cirrus SD-OCT (Spectral Domain Optical Coherence Tomography) with Enhanced depth imaging (EDI) was used to measure the MCT and CMT. The MCT is measured from the outer border of the retinal pigment epithelium (RPE) to the choroid-sclera junction at the fovea and at 500 μm intervals up to 1500 μm temporal as well as nasal to the fovea at 7 locations. (**a**) Imaging of an age and sex-matched control, (**b**) Imaging of a patient with Eales’ disease at baseline. Note the dilated choroidal vasculature in (**b**)

**Fig. 2:**
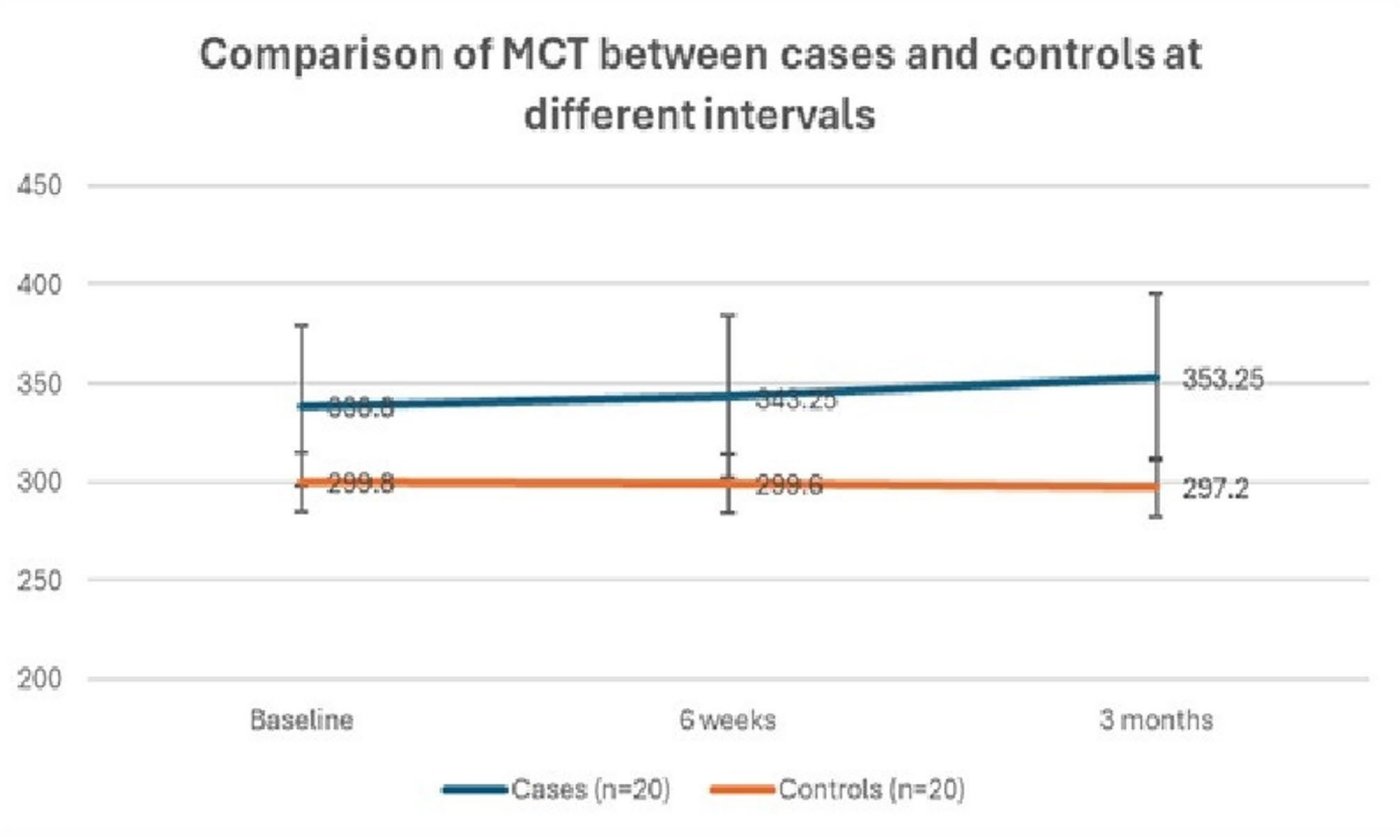
Comparison of mean choroidal thickness (MCT) (in microns) between cases and controls at different intervals

**Fig. 3:**
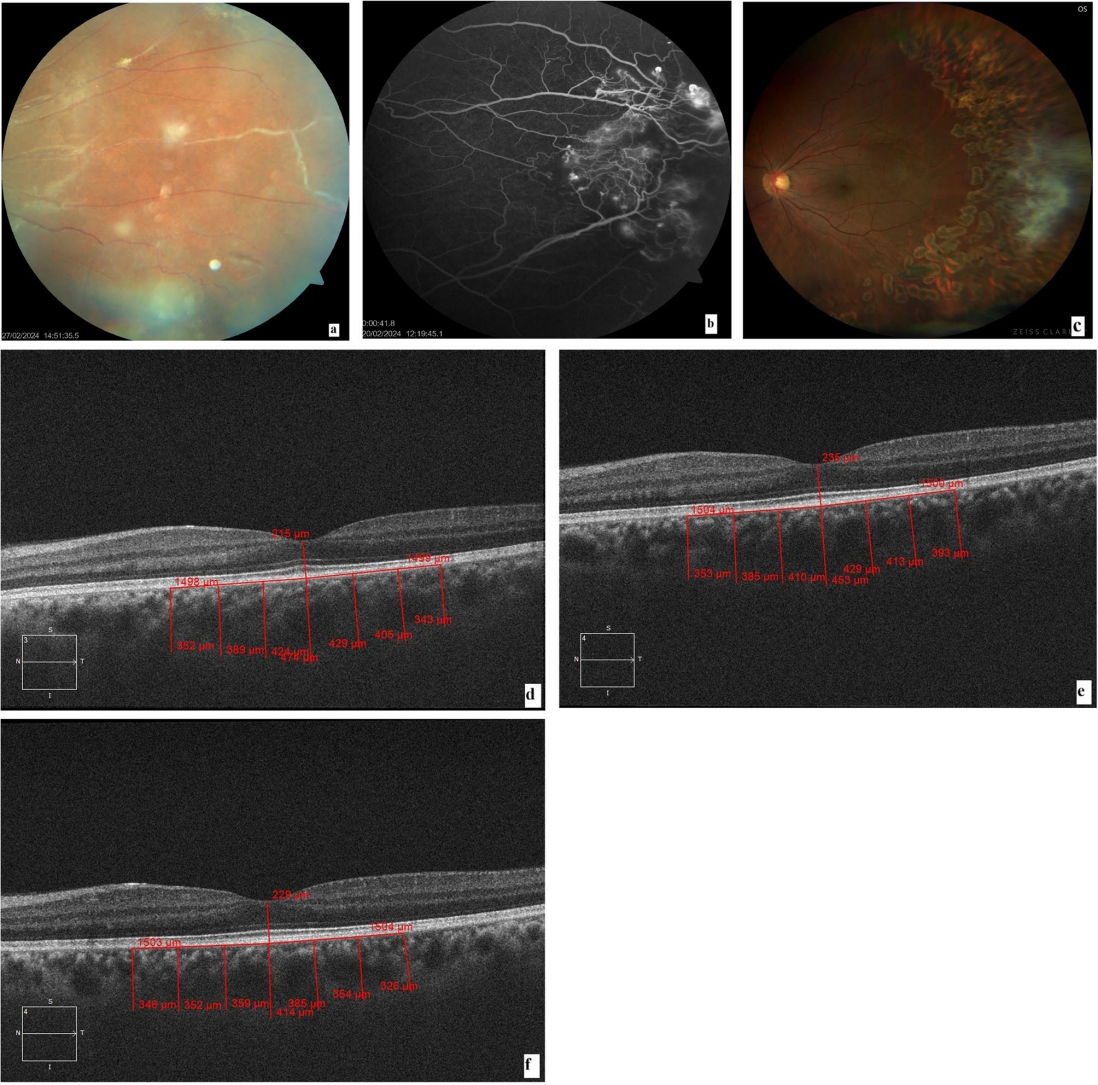
(**a**) Fundus photo shows perivascular sheathing in the inflammatory phase of Eales’ disease, (**b**) Fluorescein angiography shows neovascularization in the early arteriovenous phase and areas of capillary non-perfusion, (**c**) Fundus photo 3 months after laser photocoagulation, with regression of NVE, (**d**), (**e**), and (**f**) show Enhanced depth imaging-optical coherence tomography (EDI-OCT) images at baseline, 6 weeks, and 3 months after laser photocoagulation, respectively. These images show a persistently thickened choroid at all follow-up points

The mean baseline MCT of cases was significantly higher than the control group (p value < 0.01) (Table 2), Fig. 1. The mean MCT of the cases remained significantly higher than the controls throughout the follow-up period at 6 weeks and 3 months post laser photocoagulation (*p* < 0.01), Fig. 2. The cases showed a slight increase in MCT at 6 weeks and 3 months after laser photocoagulation, but this change was not statistically significant. Figure 3 shows fundus photos and EDI-OCT images of a patient with Eales’ disease.

**Table 2:** Comparison of mean choroidal thickness (in microns) between cases and controls

	Cases (*n* = 20)	Controls (*n* = 20)	*p*-value
Baseline	338.80 ± 46.74	299.80 ± 20.69	**< 0.01**
6 weeks	343.25 ± 44.79	299.60 ± 20.05	**< 0.01**
3 months	353.25 ± 29.25	297.20 ± 24.33	**< 0.01**
Mean change from baseline to 6 weeks	-4.45 ± 24.65	0.20 ± 0	0.49
Mean change from baseline to 3 months	-14.55 ± 43.36	2.60 ± 10.61	0.49
p-value	0.17	0.54	

Unpaired t-test was used for intergroup comparison, while Repeated Measures ANOVA was used for intragroup comparison

The mean baseline CMT of cases and controls was not statistically different (Table 3). Cases showed a small but significant increase in mean CMT 6 weeks after laser photocoagulation (*p* < 0.01).

**Table 3:** Comparison of central macular thickness (in microns) between cases and controls

	Cases (*n* = 20)	Controls (*n* = 20)	*p*-value
Baseline	240.5 ± 30.90	253.40 ± 23.52	0.10
6 weeks	258.20 ± 36.44	247.25 ± 22.79	0.43
3 months	247.40 ± 32.25	249.65 ± 24.01	0.60
Mean Change from baseline to 6 weeks	17.70 ± 20.27	-6.15 ± 10.77	**< 0.01**
Mean Change from baseline to 3 months	6.90 ± 31.74	-3.75 ± 9.98	0.05
Overall p-value	**0.04**	**0.03**	
Intragroup p-value from baseline to 6 weeks (post-hoc)	**< 0.01**	**0.02**	
Intragroup p-value from baseline to 3 months (post-hoc)	0.21	0.11	

Unpaired t-test was used for intergroup comparison, while Repeated Measures ANOVA was used for intragroup comparison at different time intervals, followed by a post hoc test

The mean logMAR BCVA of the cases and control groups was similar at all observation intervals. There was no significant change from baseline at the 3rd month in either group (*p* = 0.23 and 0.12, respectively).

## Discussion

The eye is considered to be an immune-privileged tissue owing to the blood-ocular barrier, the blood-retinal barrier, intraocular immune-modulators, and lack of intraocular lymphatics. However, when these barriers are breached in response to an alloantigen, the tolerance to self-antigens is affected. The eye may be at a greater risk of the resultant damage because its natural defences are more easily breached compared to a fully immunocompetent tissue, which is otherwise able to reject foreign antigens and mount a proportionate immune response [10]. The resultant intraocular inflammation involves the highly vascular uveal tissue and/or the retinal periphery. The latter has been found to host dendritic (antigen-presenting) cells [10]. Though the uvea is divided into three zones, namely the iris, ciliary body, and the choroid, inflammation in one often spills over into the other owing to anatomic continuity. Structures adjacent to the uveal tissue, namely the retina, optic nerve, vitreous, and sclera, may also be affected [11]. The clinical implication of this is that adjacent intraocular structures are inevitably affected in inflammatory conditions. For instance, though Eales’ disease is believed to involve mainly the peripheral retinal veins, patches of deep choroiditis along the inflamed veins have been documented, in various stages of development and healing [12].

The choroid and choroidal vasculature have been difficult tissues to image by optical methods, owing to the blocking effects of the retinal pigment epithelium. While indocyanine green angiography has been extensively used to study the same, this modality suffers from certain drawbacks. It is an invasive test with the inherent risks of drug reaction, and high-quality images are difficult to obtain owing to the low fluorescence intensity of the dye [13]. Advances in OCT technology, like SD-OCT with EDI and swept source OCT, allow fast and non-invasive study of the choroidal thickness, vasculature, and volume [14]. Despite developments in choroidal imaging, most studies have focused on pathologies associated with the pachychoroid spectrum, panuveitis (Vogt-Koyangi-Harada syndrome, Behcet disease), and posterior uveitis [15, 16]. There have been relatively fewer studies on choroidal characteristics in uveitis, where the choroid is not the primary site of inflammation- Eales’ disease, anterior uveitis, and Fuch’s uveitis syndrome [4, 17–19].

Eales’ is most commonly prevalent in Asia, particularly in the Indian subcontinent [1]. The present study was done to evaluate choroidal thickness in this idiopathic recurrent retinal periphlebitis (eponymously known as Eales’ disease) at presentation and after oral steroids and laser photocoagulation of the ischemic area. Since the natural course of Eales’ disease could be either temporary or complete remission or a course of relentless progression [20], we wanted to study the response of the choroid to treatment. Similar to the results in a study by Kumar et al. [4], baseline MCT was significantly higher than age and sex-matched healthy controls. Healthy young Indian adults have a mean sub-foveal choroidal thickness of 280.1 ± 46.5µ [14]. This increase in MCT may be due to spillover of inflammation to the choroid and/or a response of the choroidal vessels to increased vascular endothelial growth factor (VEGF) levels secondary to ischemia. What was unexpected was the finding that MCT failed to reduce after treatment, even when the clinical evaluation suggested disease quiescence. This suggests ongoing subclinical autoimmune activity for a longer period. The study by Kumar et al. in patients with idiopathic retinal vasculitis was a cross-sectional one, without any follow-up [4].

Interestingly, CT has been reported to increase with all kinds of uveitis, and decrease or become thin after resolution/ in the chronic stage [15–19, 21, 22]. Yamamoto-Rodríguez et al. found that choroidal thinning and improved vision were associated with treatment in 23 patients with VKH, followed up for 3 years [21]. Kim and colleagues studied the change in CT in Behcet’s disease, where the mean interval between the acute active and quiescent phases was 7.04+/-3.74 months [22]. Yan H et al. studied the long-term effect of inactive uveitis on CT. They studied both anterior (mean disease duration 11.3 years) and posterior uveitis (duration not mentioned, but two illustrative cases had a duration of 8 years and 22 years, respectively) [18].

Increased MCT has also been documented in patients with venous occlusion and diabetic retinopathy, which reduces/returns to normal levels after intravitreal anti-VEGF injections and laser [23, 24]. PRP reduces choroidal vascular permeability or causes atrophy of choroidal vessels over 12 weeks [25]. On the other hand, the MCT may increase significantly in the macular area immediately after PRP due to a redistribution of choroidal blood flow, which may be critical for retinal metabolism [26]. Since very few uveitic entities other than Eales’ disease require PRP, the effect of this treatment modality on CT has not been studied. Persistently increased MCT could also be due to the effect of inflammatory cytokines, like tumor necrosis factor (TNF) alpha, which have been found to be significantly elevated in Eales’ disease [27]. Since the pathology is possibly due to an exaggerated immune response to mycobacterial proteins, even in the absence of active infection, a longer period of inflammation is not surprising in the absence of continuous immune suppression. Another counterintuitive mechanism could be a steroid-induced activation of mineralocorticoid receptors resulting in dilatation and hyperpermeability of the choroidal vessels, akin to that in chronic serous chorioretinopathy [28]. But it can be argued that the steroid-induced control of inflammation in all other types of uveitis decreases the MCT. However, the increased MCT did not affect visual acuity, which did not change significantly from the baseline. The clinical significance of a persistently thickened choroid is not known and needs long-term studies to see if this is an indicator of risk of recurrence, or if the MCT takes a longer time to reduce to normal values. A detailed deliberation is needed to decide whether this subclinical evidence of ongoing inflammation warrants prolonged systemic/periocular steroids/other immune suppression, with their inherent associated side-effects. Though macular involvement in Eales’ disease has been described [29], none of our patients demonstrated evidence of maculopathy. The CMT in our patients with Eales’ disease was found to be similar to controls. This may be because central retinal periphlebitis is much rarer than peripheral involvement in Eales’, and thus the macula is spared.

Limitations of this study include a small sample size (due to a low incidence of the disease-approximately 1 in 135–200 ophthalmic patients) [30] and a relatively short follow-up period. It would be interesting to know the time taken for MCT to decrease. Moreover, since Eales’ disease may be asymptomatic for a significant amount of time, and have multiple recurrences, the exact duration of the disease cannot be elicited. This may be a confounding factor precluding a uniform baseline MCT. We also did not analyze the effect of the retinal area lasered on the MCT. Patients with larger areas lasered would be likely to experience more inflammation. In addition, though SD-OCT with EDI was used to study the choroid, manual demarcation of the retinal pigment epithelium and outer choroidal layer was done, and the calliper function was used for measuring the MCT. Automated segmentation and binarization methods would ensure more precise measurements of choroidal parameters. Higher resolution swept-source OCT may help identify the abnormalities in the different layers of the choroidal vasculature. Since normal MCT has a wide range and is influenced by location in the eye (posterior pole or periphery), age, sex, ethnicity, circadian rhythm, and refractive error, MCT alone may not be a sensitive indicator of choroidal abnormality [14]. Other parameters, like choroidal volume and choroidal vascularity index, would help understand the pathology better. These details may help define a new category of uveitic pachychoroid [31]. Moreover, a comparison of central Eales’ with peripheral Eales’ can further help us explore and evaluate the changes in the central choroid and macula. Since the primary focus of this study was the MCT, retinal points other than the fovea were not assessed.

The strength of our study is that this is possibly one of the earliest to evaluate the effect of laser photocoagulation in patients with Eales’ disease. Our results show choroidal thickening persisting at least till three months after the laser. There was no significant difference in BCVA between patients and sex and age-matched healthy controls, since Eales’ disease primarily affects the peripheral retinal. Future research should include larger patient cohorts and longer follow-up periods to provide more robust data on the changes in choroid and retina in patients with Eales’ disease.

## Conclusion

Secondary choroidal involvement is seen in Eales’ disease in the form of increased mean choroidal thickness. This altered state persists even after clinical resolution of the inflammation and laser photocoagulation. However, this does not appear to affect visual acuity in the short term, up to 3 months.

## Data Availability

No datasets were generated or analysed during the current study.

## Abbreviations

SD-OCT: Spectral domain-optical coherence tomography
EDI-OCT: Enhanced depth imaging optical coherence tomography
MCT: Mean choroidal thickness
CMT: Central macular thickness
ATT: Anti-tubercular therapy
BCVA: Best corrected visual acuity
FFA: Fundus fluorescein angiography
PRP: Pan retinal photocoagulation
CNP: Capillary non-perfusion
NVE: Neovascularization elsewhere
ICC: Intraclass correlation coefficient
logMar: Logarithm of the Minimum Angle of Resolution
VEGF: Vascular endothelial growth factor
TNF: Tumour necrosis factor
